# Naltrexone differentially modulates the neural correlates of motor impulse control in abstinent alcohol‐dependent and polysubstance‐dependent individuals

**DOI:** 10.1111/ejn.14262

**Published:** 2018-11-26

**Authors:** Liam J. Nestor, Louise M. Paterson, Anna Murphy, John McGonigle, Csaba Orban, Laurence Reed, Eleanor Taylor, Remy Flechais, Dana Smith, Edward T. Bullmore, Karen D. Ersche, John Suckling, Rebecca Elliott, Bill Deakin, Ilan Rabiner, Anne Lingford Hughes, Barbara J. Sahakian, Trevor W. Robbins, David J. Nutt, ICCAM Consortium, Filippo Passetti, Luca Faravelli, David Erritzoe, Inge Mick, Nicola Kalk, Adam Waldman, Shankar Kuchibatla, Venkataramana Boyapati, Antonio Metastasio, Yetunde Faluyi, Emilio Fernandez‐Egea, Sanja Abbott, Valerie Voon

**Affiliations:** ^1^ Neuropsychopharmacology Unit Centre for Psychiatry Imperial College London London UK; ^2^ Department of Psychiatry University of Cambridge Cambridge UK; ^3^ Neuroscience and Psychiatry Unit University of Manchester Manchester UK; ^4^ Department of Psychology University of Cambridge Cambridge UK; ^5^ Imanova Centre for Imaging Sciences Invicro London UK

**Keywords:** addiction, functional MRI, impulsivity, naltrexone

## Abstract

Identifying key neural substrates in addiction disorders for targeted drug development remains a major challenge for clinical neuroscience. One emerging target is the opioid system, where substance‐dependent populations demonstrate prefrontal opioid dysregulation that predicts impulsivity and relapse. This may suggest that disturbances to the prefrontal opioid system could confer a risk for relapse in addiction due to weakened ‘top‐down’ control over impulsive behaviour. Naltrexone is currently licensed for alcohol dependence and is also used clinically for impulse control disorders. Using a go/no‐go (GNG) task, we examined the effects of acute naltrexone on the neural correlates of successful motor impulse control in abstinent alcoholics (AUD), abstinent polysubstance‐dependent (poly‐SUD) individuals and controls during a randomised double blind placebo controlled fMRI study. In the absence of any differences on GNG task performance, the AUD group showed a significantly greater BOLD response compared to the control group in lateral and medial prefrontal regions during both placebo and naltrexone treatments; effects that were positively correlated with alcohol abstinence. There was also a dissociation in the positive modulating effects of naltrexone in the orbitofrontal cortex (OFC) and anterior insula cortex (AIC) of the AUD and poly‐SUD groups respectively. Self‐reported trait impulsivity in the poly‐SUD group also predicted the effect of naltrexone in the AIC. These results suggest that acute naltrexone differentially amplifies neural responses within two distinct regions of a salience network during successful motor impulse control in abstinent AUD and poly‐SUD groups, which are predicted by trait impulsivity in the poly‐SUD group.

## Introduction

Impulsive behaviour has been implicated in the development and maintenance of addiction disorders (Verdejo‐Garcia *et al*., 2008; Volkow & Baler, 2012). Impulsivity likely arises due to a breakdown in mechanisms underlying prefrontal‐mediated inhibitory control (Goldstein & Volkow, 2002; Kaufman *et al*., 2003; Lubman *et al*., 2004) that may affect the ability to restrain actions related to continued substance use. Inhibitory control deficits have been commonly observed in various addiction populations (Fillmore & Rush, 2002; Fu *et al*., 2008; Rubio *et al*., 2008; Luijten *et al*., 2011), suggesting that the ability to inhibit impulsive actions is a core feature of addiction disorders, and a possible predictor of substance relapse (Moeller *et al*., 2001; Bowden‐Jones *et al*., 2005; Krishnan‐Sarin *et al*., 2007). Therefore, identifying neural substrates of impulse control in addiction disorders that could act as therapeutic targets for relapse prevention during abstinence remains a major challenge in human clinical neuroscience.

Pre‐clinical animal models show that the mu opioid receptor (MOR) plays a significant role in addiction (Giuliano *et al*., 2013), particularly with respect to alcohol‐seeking and consumption (Boyle *et al*., 1998; Dayas *et al*., 2007; Giuliano *et al*., 2015). Importantly, appetitive motivation for addictive substances may also occur due to disturbances in the prefrontal opioid system that could conceivably result in the recruitment of diverse and functionally opposed pathways (Baldo, 2016) that lead to a breakdown in mechanisms of inhibitory control, which promote substance relapse. Indeed, animal models have shown that the MOR promotes impulsive behaviours that are actually dissociated from alcohol or drug consumption (Olmstead *et al*., 2009; Pattij *et al*., 2009; Mahoney *et al*., 2013; Sanchez‐Roige *et al*., 2015), suggesting that opioid disturbances within prefrontal brain networks could be targets for diminishing impulsivity in addiction disorders. There is evidence for opioid dysregulation in the prefrontal cortex in human addiction disorders (Gorelick *et al*., 2005, 2008; Williams *et al*., 2007; Ghitza *et al*., 2010), with evidence that endogenous opioids may promote impulsivity in humans (Love *et al*., 2009). Clinical evidence also suggests that MOR blockade with naltrexone is effective in some impulse control disorders (Kim *et al*., 2001; Grant, 2005; Lahti *et al*., 2010; Grant *et al*., 2014), promotes abstinence in substance addiction populations (Krystal *et al*., 2001; Srisurapanont & Jarusuraisin, 2005; Grassi *et al*., 2007), possibly through the promotion of impulse control (Sanchez‐Roige *et al*., 2015). There is evidence that naltrexone can also ameliorate neural disturbances in addiction (Savulich *et al*., 2017; Morris *et al*., 2018), including those related to impulsive choice (Boettiger *et al*., 2009), pointing to its potential efficacy as a neuromodulator of impulse control. Therefore, evidence of disturbances to prefrontal endogenous opioid functioning, together with the clinical efficacy of naltrexone in treating impulse control and addiction disorders, suggests that this system may be a viable target for relapse prevention, where there are deficits related to impulsivity.

Comorbid alcohol and drug dependence may be particularly detrimental to health. Individuals who have been dependent on multiple substances report the consumption of more alcohol have a greater incidence and severity of psychiatric illness, than individuals who are exclusively alcohol‐dependent (Moss *et al*., 2015). This divergence in abuse and dependence, could conceivably, induce disparate neural responses that are a product of dependence ‘phenotype’, and that differentially respond to medications that modulate endogenous opioid functioning. Therefore, the current study investigated the effects of acute MOR blockade with naltrexone on the neural correlates of motor impulse control using a go/no‐go task (GNG) in alcoholic *and* polysubstance‐dependent individuals who were in extended abstinence. The GNG task is sensitive to the neural correlates of impulse control during heightened performance monitoring, exploiting the same prefrontal networks that are targets of the endogenous opioid system and MOR blockade with naltrexone. Therefore, we hypothesised that (i) compared to the control group, the alcoholic and polysubstance‐dependent groups would show poorer motor impulse control, (ii) compared to the control group, the alcoholic and polysubstance‐dependent groups would demonstrate disparate functional differences within prefrontal networks due to dependence ‘phenotype’, and (iii) that the alcoholic and polysubstance‐dependent groups would demonstrate disparate remediating effects on these functional differences following acute MOR blockade with naltrexone.

## Material and methods

### Participants

This was a randomised, double‐blind, placebo‐controlled multi‐centre study involving three study sites in the United Kingdom (Imperial College London, University of Cambridge and University of Manchester). For a more detailed description of the ICCAM Platform, see Paterson *et al*. (Paterson *et al*., 2015). Inclusion criteria were individuals who met DSM‐IV criteria for current or prior alcohol dependence (AUD), or alcohol plus (poly‐SUD) another substance of dependence (e.g., amphetamines, benzodiazepines, cocaine, opiates) and who were abstinent for at least 4 weeks prior to the experimental sessions. There was no upper limit for abstinence length. All participants were aged 21–64 years of age. For a full description of the cohort used in the current study, please see Nestor *et al*. (2017), which examined the same participant groups using a monetary incentive delay task (Nestor *et al*., 2017). In the current study, the AUD group was made up of 21 abstinent alcoholics, with the poly‐SUD group comprised of 25 abstinent alcoholic polysubstance‐dependent individuals (having met criteria for dependence to alcohol plus one or more other substances of dependence). The healthy control group was made up of 35 participants with no previous history of substance abuse, as assessed using the Alcohol, Smoking and Substance Involvement Screening Test (ASSIST) (WHO ASSIST Working Group, 2002) and timeline follow‐back. All participants were required to provide a negative breath alcohol test and a negative urine sample for various drugs of abuse on both experimental days (screening for the presence of amphetamines, benzodiazepines, cannabinoids, cocaine and opiates). The Mini*‐*International Neuropsychiatric Interview *(*MINI*)* (Sheehan *et al*., 1998) was administered to all participants by a trained psychiatrist to screen for the presence of Axis I psychiatric disorders that were part of the study exclusion criteria.

Exclusion criteria included (i) current use of regular prescription or non‐prescription medications that could not be stopped for the study duration, or would interfere with study integrity or subject safety (including but not limited to antipsychotics, anticonvulsants, antidepressants, disulfiram, acamprosate, naltrexone, varenicline); (ii) current primary axis I diagnosis, past history of psychosis (unless drug‐induced); (iii) current or past history of enduring severe mental illness (e.g., schizophrenia, bipolar affective disorder); (iv) other current or past psychiatric history that, in the opinion of a psychiatrist, contraindicated participation; (v) history or presence of a significant neurological diagnosis that may have influenced the outcome or analysis of the results (including but not limited to stroke, epilepsy, space occupying lesions, multiple sclerosis, Parkinson's disease, vascular dementia, transient ischaemic attack, clinically significant head injury); (vi) claustrophobia or unable to lie still in the MRI scanner for up to 90 min; (vii) presence of a cardiac pacemaker, other electronic device or other MRI contraindication, including pregnancy, as assessed by a standard pre‐MRI questionnaire. Secondary or lifetime history of depression or anxiety was permitted in both substance abusers and healthy controls as these are very common psychiatric disorders.

All participants provided written informed consent. The study was conducted in accordance with the Declaration of Helsinki. Ethical approval was obtained from West London and Gene Therapy Advisory Committee National Research Ethics Service committee (11/H0707/9) and relevant research governance and Participant Identification Centre (PIC) approvals obtained.

### Experimental visits

At the randomised placebo and naltrexone experimental visits (visits 2 or 3), an eligibility check was performed. Participants’ intervening drug use and concomitant medication were checked and participants completed alcohol breath, pregnancy and urine drugs of abuse screening tests. Cigarette smokers in all groups smoked *ad lib* approximately 60 min prior to scanning in order to avoid the potential confounds of withdrawal and/or craving during scanning.

### Medications

Drug preparation, labelling and packaging were performed by UCLH Pharmacy Manufacturing Unit. Placebo was Vitamin C (100 mg, supplier: Sigma, manufacturer: Norbrook) and naltrexone (50 mg Nalorex^®^ ‐ manufacturer ‐ Bristol‐Myers Squibb) were prepared and packaged according to Investigational Medicinal Product guidelines. The maximum naltrexone plasma concentration after an acute 50 mg dose occurs between 0.5 and 3 h (Meyer *et al*., 1984). Therefore, participants were dosed 2 h prior to each experimental scan session to ensure high MOR occupancy during testing. Naltrexone and placebo medications were supplied in identical white opaque bottles and administered by independent nursing staff, such that both researcher and participant remained blinded.

### Go/no‐go (GNG) task

The GNG task (Garavan *et al*., 2003) consisted of alternating target stimuli (the letters *X* and *Y*), each of which were presented for 900 milliseconds (ms), immediately followed by a 100 ms inter‐stimulus interval. Participants were required to make a response (on a hand‐held key pad) as quickly as possible to each stimulus (‘go’ trials). Participants were additionally required to inhibit their response (‘stop’ trials) when the target stimuli did not alternate (i.e. the second of two identical, successively presented target stimuli – e.g., respond to all stimuli except the fifth in the sequence XYXYYX). Participants were required to immediately recommence responding to the alternating stimuli following the presentation of a ‘stop’ stimulus. There were a total of 250 stimuli presented in each run of the task, of which 30 were ‘stop’ trials. Participants completed a total of two runs of the task, with each run lasting 262 s. Dependent measures for the task were the mean ‘go’ and ‘stop’ percentage accuracy, together with mean ‘go’ and mean ‘error’ response times (ms). The task was programmed using E‐Prime (Psychology Software Tools, Pittsburgh, USA).

### Functional MRI (fMRI) data acquisition

For a more comprehensive description of data acquisition across the three sites, please see McGonigle *et al*. (McGonigle *et al*., 2017). Briefly, all centres operated MRI machines with a main magnetic field of 3 tesla (T). Centres in London and Cambridge operated nominally identical 3T Siemens Tim Trio systems running the syngo MR B17 software with a Siemens 32 channel receive‐only phased‐array head coil. The Manchester centre operated a 3T Philips Achieva running version 2.6.3.5 software and an 8 element SENSE head coil. For anatomical images, 160 high‐resolution T1‐weighted anatomic MPRAGE axial images (FOV 256 mm, thickness 1.0 mm, voxel size 1.0 × 1.0 × 1.0) were acquired (total duration 303 s). Functional data were acquired using a T2* weighted echo‐planar imaging sequence collecting 36 non‐contiguous (0% gap) 3.0 mm axial slices covering the entire brain (TE = 31 ms, TR = 2000 ms, FOV 225 mm, 64 × 64 mm matrix size in Fourier space). Each run of the GNG task produced a total of 161 volumes of functional MRI data.

### GNG fMRI data analyses

Data pre‐processing and statistical analysis were conducted using FEAT (fMRI Expert Analysis Tool) from the FMRIB Software Library (www.fmrib.ox.ac.uk/fsl). Pre‐statistical processing was as follows: motion correction utilising FMRIB's Linear Image Registration Tool (MCFLIRT; non‐brain matter removal using Brain Extraction Tool (BET); spatial smoothing with a 5‐mm full‐width half maximum Gaussian kernel; mean‐based intensity normalisation; non‐linear high‐pass temporal filtering (Gaussian‐weighted least squares straight line fit, with sigma = 25.0 s). The six rigid body movement parameters were also included as regressors in the model in FSL FEAT.

For each participant, first level whole brain mixed‐effects analyses were performed by separately modelling the ‘stop’ and ‘error’ trials (stick function regressors were convolved with the haemodynamic response function). The ‘go’ trials acted as the baseline for both the ‘stop’ and ‘error’ measures, as this period reflects tonic task‐related processes of the GNG task. Therefore, all reported differences are for activation changes vs. the baseline. Registration was conducted through a two‐step procedure, whereby EPI images were first registered to the high‐resolution T1 structural image, then into standard (Montreal Neurological Institute, MNI avg152 template) space, with 12‐parameter affine transformations.

Three (Group: AUD vs. poly‐SUD vs. control) by two (Drug: placebo vs. naltrexone) whole brain cluster‐based repeated measures ANOVA analyses were conducted as part of a higher‐level mixed‐effects analysis on the ‘stop’ and ‘error’ activation measures. These higher‐level analyses were conducted using FLAME (FMRIB's Local Analysis of Mixed Effects). Cluster (Gaussianised F) statistical images were determined by *Z *>* *2.3 with a corrected cluster significance threshold of *P *<* *0.05. This ANOVA analysis produced a total of three (i.e. drug effect, group effect, drug × group interaction) zf statistical images for the ‘stop’ and ‘error’ measures. Owing to scanner differences across the three sites, we used site as a covariate in the cluster‐based repeated measures ANOVA analyses.

### Other statistics

Between‐groups demographics (see Table S1.) were examined using Kruskal*–*Wallis (gender distribution and drug order) or one‐way ANOVA analyses. For analyses conducted on the GNG behavioural measures, three (Group: AUD vs. poly‐SUD vs. control) by two (Drug: placebo vs. naltrexone) repeated measures ANOVA analyses were conducted. We also conducted a three (Group: AUD vs. poly‐SUD vs. control) by two (Drug: placebo vs. naltrexone) repeated measures ANOVA on an index of the relative ‘impulsivity’ value (RIV). This value is based on the ratio of mean reaction times on ‘go’ trials compared to that on ‘error’ trials – i.e. (“go” rt/“error” rt. Here a value >1 reflects a higher relative value of impulsive responding when a participant commits an ‘error’ on a no‐go trial. The mean BOLD signal change for each repeated measures zf statistical cluster was also extracted in order to conduct follow‐up *post hoc* analyses. These follow‐up analyses used either Bonferroni pair‐wise or independent *t*‐test analyses to examine group and interaction effects respectively. Correlations between demographics or baseline questionnaire measures and the mean BOLD signal change from the zf statistical clusters were assessed using Pearson's correlations. Owing to the variability in alcohol abstinence in both the AUD and poly‐SUD groups, data were Log (10) transformed prior to correlation analyses, to eliminate the influence of positive skew. These analyses were all conducted using the Statistical Package for the Social Sciences (SPSS Inc., Chicago).

### Baseline questionnaires

We assessed self‐reported impulsivity at baseline (visit 1) using both the Barratt Impulsivity scale (BIS‐11) (Patton *et al*., 1995) and the UPPS‐P scale (Whiteside & Lynam, 2001). These measures were acquired to examine if reported baseline impulsivity could predict the delta (naltrexone minus placebo) mean BOLD signal change in brain regions of the AUD and poly‐SUD groups during ‘stop’ trials of the GNG task. Only the composite score on both these questionnaires was used when conducting the correlations.

## Results

### Demographics

The groups significantly differed on most of the measures reported (see Table S1 in supporting information for a more detailed description of demographic group differences). The AUD group was significantly older than the poly‐SUD group; the poly‐SUD group reported significantly less years of education, and had a lower estimated IQ, than the control group; the poly‐SUD group had significantly more cigarette use than the control group, and reported significantly more cannabis use than both controls and the poly‐SUD group. The AUD and poly‐SUD groups, however, did not significantly differ on cigarette use (pack years) or alcohol abstinence, but the AUD group did have significantly more alcohol exposure than the poly‐SUD group. Importantly, the three groups did not differ significantly on drug treatment order (χ^2^ = 0.48, df = 2, *P *=* *0.78) during the study.

### GNG performance

**Figure 1:**
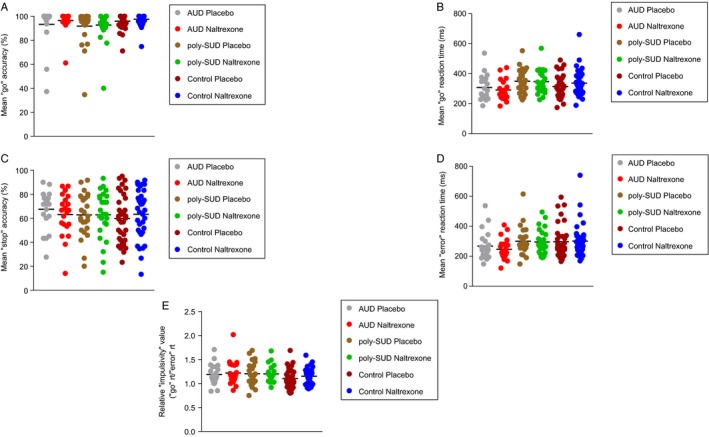
Showing GNG task performance in the AUD, poly‐SUD and control groups during the placebo and naltrexone sessions for (A) mean percentage ‘go’ accuracy; (B) mean ‘go’ reaction time; (C) mean ‘stop’ accuracy, (D) mean ‘error’ reaction time and E) the relative ‘impulsivity’ value (RIV). Data were analysed using a three (Group: AUD vs. poly‐SUD vs. control) by two (Drug: placebo vs. naltrexone) repeated measures ANOVA. Data are expressed as means.

Figure 1A below shows the mean ‘go’ accuracy (%) for the AUD, poly‐SUD and control groups during the placebo and naltrexone sessions. A three (Group: AUD vs. poly‐SUD vs. control) by two (Drug: placebo vs. naltrexone) repeated measures ANOVA revealed no effect of drug (*F *=* *2.52; df = 1, 78; *P *=* *0.11), group (*F *=* *1.76; df = 2, 78; *P *=* *0.17) or a drug × group interaction (*F *=* *0.31; df = 2, 78; *P *=* *0.73). The same analysis for mean ‘go’ reaction time (ms) revealed no effect of drug (*F *=* *0.03; df = 1, 78; *P *=* *0.85), but did reveal trends for a group effect (*F *=* *2.52; df = 2, 78; *P *=* *0.087), and a drug × group interaction (*F *=* *2.88; df = 2, 78; *P *=* *0.062 – Fig. 1B). For mean ‘stop’ accuracy (%), there was no drug (*F *=* *0.039; df = 1, 78; *P *=* *0.84), or group effect (*F *=* *0.27; df = 2, 78; *P *=* *0.75), but there was a trend for a drug × group interaction (*F *=* *2.64; df = 2, 78; *P *=* *0.078 – Fig. 1C). For mean ‘error’ reaction time (ms), there was no effect of drug (*F *=* *0.62; df = 1, 78; *P *=* *0.43), group (*F *=* *1.73; df = 2, 78; *P *=* *0.18) or a drug × group interaction (*F *=* *0.63; df = 2, 78; *P = *0.53 – Fig. 1D). Finally, for the RIV index, there was no effect of drug (*F *=* *1.06; df = 1, 78; *P *=* *0.31), group (*F *=* *1.93; df = 2, 78; *P *=* *0.15) or a drug × group interaction (*F *=* *0.44; df = 2, 78; *P = *0.64 – Fig. 1E).

### Functional MRI

**Figure 2:**
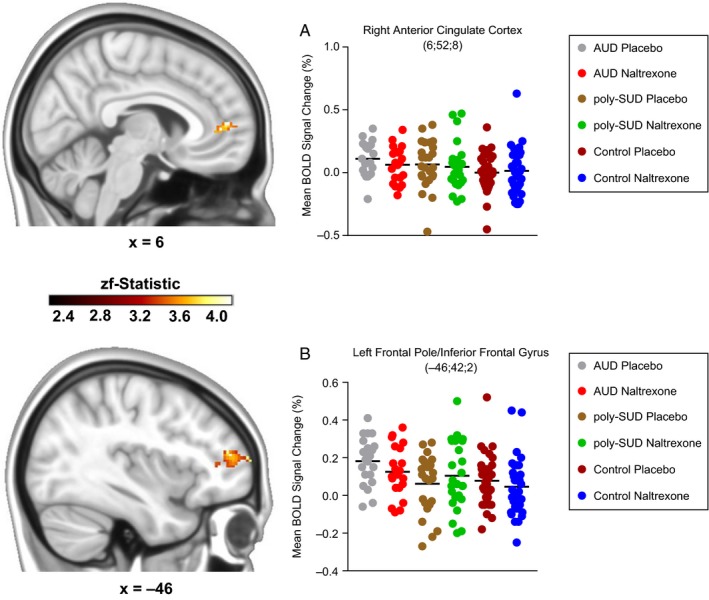
Three (Group: AUD vs. poly‐SUD vs. control) by two (Drug: placebo vs. naltrexone) cluster‐based repeated measures ANOVA showing a group effect in (A) the right anterior cingulate cortex (AUD>control, *P *=* *0.041, *post hoc* Bonferroni) and (B) the left frontal pole/inferior frontal gyrus (AUD>control, *P *=* *0.005, *post hoc* Bonferroni). The scale represents the colour (from dark to light yellow) of the cluster voxels corresponding to the increasing zf‐statistic that came from the initial cluster‐based repeated measures ANOVA. Data are expressed as means. Coordinates are represented in Montreal Neurological Institute (MNI) space.

All three groups demonstrated statistically significant activation patterns across lateral and midline frontal regions during the placebo and naltrexone challenges for ‘stops’ at a whole brain level – although these activation changes did appear more robust in the AUD group (see Figs S1 and S2). A three (Group: AUD vs. poly‐SUD vs. control) by two (Drug: placebo vs. naltrexone) whole brain cluster‐based repeated measures ANOVA revealed a significant main effect of group in the right anterior cingulate cortex (280 voxels; *x* = 6; *y* = 52; *z* = 8; zf_stat_ = 3.55; *P *=* *0.003; AUD_ _> control, *P *=* *0.041_*Bonferroni*_ ‐ Fig. 2A); left frontal pole/inferior frontal gyrus (366 voxels; *x* = −46; *y* = 42; *z* = 2; zf_stat_ = 4.42; *P *=* *0.003; AUD > control, *P *=* *0.005_*Bonferroni*_ – Fig. 2B); and right inferior frontal gyrus (491 voxels; *x* = 46; *y* = 30; *z* = 4; zf_stat_ = 3.48; *P *=* *0.002; AUD > control, *P *=* *0.049_*Bonferroni*_).

**Figure 3:**
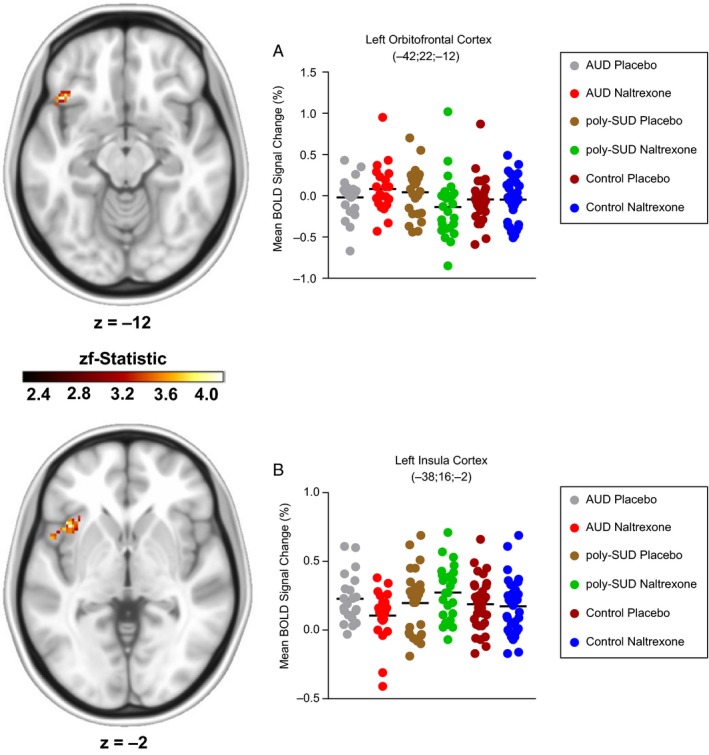
Three Group (Group: AUD vs. poly‐SUD vs. control) by two (Drug: placebo vs. naltrexone) cluster‐based repeated measures ANOVA showing a drug × group interaction in (A) the left orbitofrontal cortex (AUD>poly‐SUD,* P *=* *0.033, *post hoc* Independent *t*‐test) and (B) the left anterior insula cortex (poly‐SUD > AUD,* P *=* *0.005, *post hoc* Independent *t*‐test). The scale represents the colour (from dark to light yellow) of the cluster voxels corresponding to the increasing zf‐statistic that came from the initial cluster‐based repeated measures ANOVA. Data are expressed as means. Coordinates are represented in Montreal Neurological Institute (MNI) space.

The same analysis also revealed a significant drug × group interaction in the left orbitofrontal cortex (127 voxels; *x* = −42; *y* = 22; *z* = −12; zf_stat_ = 4.1; *P *=* *0.006 – Fig. 3A) and left anterior insula cortex (162 voxels; *x* = −38; *y* = 16; *z* = −2; zf_stat_ = 4.04; *P *=* *0.005 – Fig. 3B). Follow‐up independent *t*‐test analyses on the left orbitofrontal cortex cluster revealed that the AUD group demonstrated a significantly greater activation change compared to the poly‐SUD group on naltrexone (*t*(44) = 2.2, *P *=* *0.033), with a trend for a greater activation change compared with the control group (*t*(54) = 1.7, *P *=* *0.096). Analyses for the left anterior insula cluster revealed that the poly‐SUD group demonstrated a significantly greater activation change compared with the AUD group (*t*(54) = 2.9, *P *=* *0.005) on naltrexone, with a trend for a greater activation change compared to the control group (*t*(58) = 1.9, *P *=* *0.05). There was no effect of drug for ‘stops’, and there was no effect of drug, group or a drug × group interaction for ‘errors’ for the same whole brain cluster‐based repeated measures ANOVA.

### Correlations

**Figure 4:**
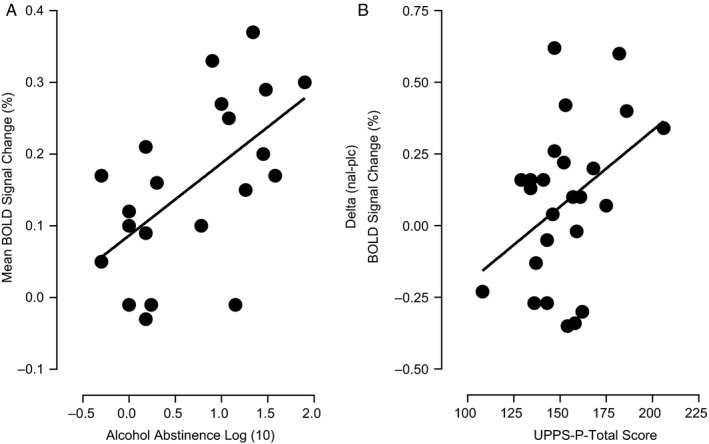
Showing significant positive correlations between (A) alcohol abstinence (mths) and the mean (of placebo and naltrexone) left frontal pole/inferior frontal gyrus ‘stop’ BOLD signal change (*r*(19) = 0.58, *P *=* *0.005) in the AUD group, and (B) total baseline UPPS‐P score and the delta (naltrexone minus placebo) ‘stop’ BOLD signal change in the left anterior insula cortex cluster (*r*(23) = 0.40, *P *=* *0.027) in the poly‐SUD group.

There were two significant correlations observed. Alcohol abstinence (months) was significantly positively correlated with the mean (of placebo and naltrexone) left frontal pole/inferior frontal gyrus ‘stop’ BOLD signal change (*r*(19) = 0.58, *P *=* *0.005) in the AUD group (Fig. 4A) – the same cluster where this group was significantly higher than the control group across drug visits. The total baseline UPPS‐P score was also found to be significantly positively correlated with the delta (naltrexone minus placebo) ‘stop’ BOLD signal change in the anterior insula cortex (*r*(23) = 0.40, *P *=* *0.027) of the poly‐SUD group (Fig. 4B) – the same cluster showing the drug × group interaction effect in the poly‐SUD group.

## Discussion

The current study compared the effects of naltrexone on the neural correlates of motor impulse control in AUD, poly‐SUD and healthy controls using a GNG task. In the absence of any drug or group effects on GNG task performance, the AUD group demonstrated a significantly greater neural response, during both placebo and naltrexone treatments, in lateral and medial prefrontal regions compared to controls during response inhibition. Activation differences were significantly correlated with alcohol abstinence in the AUD group. We further report an apparent disparity in the positive modulating effects of acute naltrexone in the orbitofrontal cortex (OFC) and anterior insula cortex (AIC) of the AUD and poly‐SUD groups respectively, possibly pointing to a differential neural effect in these populations during motor impulse control. The AIC naltrexone effect during motor impulse control was also positively correlated with reported trait impulsivity in the poly‐SUD group.

### Greater neural effects in the AUD group during motor impulse control

The monitoring of one's behaviour during abstinence may be especially important when there is a need to override impulses, particularly those that might induce relapse (Garavan & Stout, 2005). Furthermore, treatment adherence may rely upon addicted individuals exercising greater prefrontal control over drug‐seeking behaviours (Goldstein & Volkow, 2002; Everitt *et al*., 2008). There is evidence that substance‐dependent groups in long‐term abstinence demonstrate increased prefrontal activation during tasks that exploit cognitive control (Nestor *et al*., 2011; Connolly *et al*., 2012; Kroenke *et al*., 2015). The current study showed that the AUD group, who were long‐term abstinent alcoholics, demonstrate significantly greater activation, compared to controls, in the anterior cingulate cortex (ACC) and bilateral frontal pole/inferior frontal gyrus (FP/IFG) during motor impulse control.

The ACC has been implicated in the execution of urgent inhibitions, reflecting heightened performance monitoring (Garavan *et al*., 2002; Botvinick *et al*., 2004; Ridderinkhof *et al*., 2004); with functional disturbances in addiction populations in this region having previously been reported (Goldstein & Volkow, 2002; Peoples, 2002; Volkow *et al*., 2002). Similarly, the FP/IFG are involved in higher order cognition (Burgess *et al*., 2007), particularly response inhibition (Swick *et al*., 2008), and are co‐activated with regions such as the ACC during cognitive control (Gilbert *et al*., 2010). The amplified response in our group of abstinent alcoholics, therefore, may lend further credence to the supposition of emerging neurocognitive adaptations within prefrontal circuitry that promote long‐term abstinence, and possibly, protect against relapse in this population. Another possibility is that the greater activation patterns observed in the AUD group represent deficits in cortical grey matter volume (GMV) in the medial and lateral prefrontal regions. There is evidence that patients with AUD have density (Thayer *et al*., 2016) and volume deficits (Medina *et al*., 2008; Trick *et al*., 2014; Xiao *et al*., 2015; Gropper *et al*., 2016) in the prefrontal cortex, which may also be correlated with impulse control (Wiers *et al*., 2015; Gropper *et al*., 2016; Grodin *et al*., 2017), and predict future relapse (Cardenas *et al*., 2011). This raises the possibility that the effects observed in the AUD group represent some compensatory mechanism by which prefrontal networks expend excessive energy to support cognitive control processes in abstinence due to structural brain deficits caused by alcohol abuse. The correlation between alcohol abstinence and FP/IFG activation in the AUD group, however, may discount this possibility.

Addiction disorders, such as AUD, are generally associated with impairments in impulse control (Smith *et al*., 2014). There is a mixed literature regarding the neural correlates of impulse control in AUD, however. The reported activation pattern observed in the FP of the AUD group in the present study, for example, may concur with previous results in abstinent alcoholics during impulse control (Li *et al*., 2009), who similarly found increased activation in the bilateral prefrontal cortex using a stop signal task (SST). Another study has shown greater cortical activation patterns in AUD (Hu *et al*., 2015), but not in the prefrontal cortex, and in the presence of poorer impulse control. Other studies, however, have not reported group differences on the neural substrates of impulse control in AUD, instead showing associations between diminished prefrontal responses and harmful alcohol use (Hu *et al*., 2016) or no differences at all (Taylor *et al*., 2016), despite high self‐report measures of impulsivity. These differences in impulse control and its neural correlates in AUD across studies may reflect contrasting durations of abstinence at the time of testing that represent differences in the remission of impulsivity in AUD (Sinha, 2011; Naim‐Feil *et al*., 2014).

### Disparate neural effects of naltrexone in the AUD and poly‐SUD groups during motor impulse control

The current study observed a divergent modulating effect of naltrexone in the OFC and AIC of the AUD and poly‐SUD groups respectively. The effects of naltrexone on the neural correlates of motor impulse control appeared to be confined to the left OFC in the AUD group, who showed a significantly greater response compared to the poly‐SUD group during naltrexone treatment. The OFC is considered to be a critical frontal region in the suppression of behaviours (Horn *et al*., 2003; Chikazoe *et al*., 2009), possibly through the updating of ‘salient’ events (Torregrossa *et al*., 2008). The OFC also contains a high number of MORs (Gorelick *et al*., 2005), possibly making it a target for the modulating effects of naltrexone in this region under conditions of motor impulse control. Naltrexone, which is currently licensed for alcohol dependence and impulse control disorders such as gambling, may remediate neural disturbances by restoring some balance within prefrontal circuitry that is critical for the suppression of impulsive responding in addiction disorders. Acute naltrexone has been shown to enhance activation in the lateral OFC of abstinent alcoholics during a delay discounting task (Boettiger *et al*., 2009), for example, providing some evidence for its neural effects during impulse control. The observed acute effects of naltrexone in the current study may further represent an enhancement of OFC functioning, possibly through its updating of ‘salient’ (no‐go) events that promotes behavioural inhibition.

The effects of acute naltrexone in the poly‐SUD group, by contrast, were observed in the left AIC, where they showed a significantly greater response compared to the AUD group during naltrexone treatment. The AIC responds under conditions of inhibitory control (Garavan *et al*., 1999), possibly through the detection (rather than inhibition) of ‘salient’ (no‐go) events (Uddin, 2015) involving behavioural risk (Preuschoff *et al*., 2008) and the requisite of avoidance behaviour (Krawitz *et al*., 2010). The insula cortex contains a high number of MORs (Zubieta *et al*., 2005), also making it a target for the modulating effects of naltrexone. We previously reported that the same poly substance group showed a diminished response in the left AIC during acute naltrexone in response to missed rewards on a monetary incentive delay task (Nestor *et al*., 2017), suggesting a blunting of error‐related signalling by naltrexone. The current study, however, suggests an opposing effect of naltrexone in the AIC, possibly by amplifying the detection of no‐go events under the demands of heightened performance monitoring. We additionally found that self‐reported trait impulsivity in the poly‐SUD group was significantly correlated with the delta signal in the AIC. This appears to suggest that the greatest neural effects of naltrexone in the AIC, under conditions requiring motor impulse control, were in those with highest reported levels of trait impulsivity prior to naltrexone treatment.

The apparent disparate modulating effects of naltrexone in the alcohol and poly substance groups, within two distinct regions of a salience network during the exploits of successful motor impulse control, however, remains unclear. These differences may have arisen due to the ‘compounding’ toxic effects of chronic alcohol and drug dependence in the poly‐SUD group. The poly‐SUD group was made up a heterogeneous sample, predominantly reporting previous dependence on amphetamines, cocaine and opiates in addition to alcohol. These long‐term toxic effects, therefore, may have conferred a response to MOR blockade in a region that is involved in the detection (rather than inhibition) of ‘salient’ events, possibly due to acute changes in the awareness of interoceptive (i.e. bodily) states (Critchley *et al*., 2004) during the demands of cognitive control. The OFC, conversely, is considered to be a frontal region critical in the suppression of behaviours, perhaps suggesting that acute MOR blockade with naltrexone in the AUD group remediates functioning in a region typically exploited during executive functioning. While speculative, these results may point, tentatively, towards a poly substance effect in the AIC that needs to be addressed in a larger sample.

There were a number of limitations of the current study, which included a lack of complete matching of groups with respect to age, cannabis and cigarette use, anxiety and mood measures. We did not use these metrics as covariates for any of our fMRI analyses, and therefore, cannot unequivocally dismiss their potential influence on the group and interaction effects reported herein. Moreover, we did not thoroughly assess alcohol and drug craving at either session in the AUD and poly‐SUD groups. This may have conceivably influenced the observed effects of naltrexone with respect to the neural correlates of cognition.

## Conclusion

In summary, the current study set out to examine the acute modulating effects of MOR blockade upon the behavioural and neural correlates of motor impulse control in abstinent alcoholic and polysubstance‐dependent individuals. Here we have provided evidence that naltrexone differentially amplifies neural responses within two distinct regions of a salience network during the demands of successful motor impulse control, in two independent addiction populations who are in extended abstinence. Exaggerated responses in abstinent alcoholics may further support the notion of emerging neurocognitive adaptations within prefrontal networks that promote long‐term abstinence, and possibly, protect against relapse.

## Conflict of interests

The authors declared the following potential conflicts of interest with respect to the research, authorship, and/or publication of this article: David J Nutt is an advisor to British National Formulary, MRC, General Medical Council, Department of Health, is President of the European Brain Council, past President of the British Neuroscience Association and European College of Neuropsychopharmacology, chair of the Independent Scientific Committee on Drugs (UK), is a member of the International Centre for Science in Drug Policy, advisor to Swedish government on drug, alcohol and tobacco research, editor of the Journal of Psychopharmacology, sits on advisory Boards at Lundbeck, MSD, Nalpharm, Orexigen, Shire, has received speaking honoraria (in addition to above) from BMS/Otsuka, GSK, Lilly, Janssen, Servier, is a member of the Lundbeck International Neuroscience Foundation, has received grants or clinical trial payments from P1vital, MRC, NHS, Lundbeck, has share options with P1vital, has been expert witness in a number of legal cases relating to psychotropic drugs, and has edited/written 27 books, some purchased by pharmaceutical companies. Trevor W Robbins has research grants with Eli Lilly and Lundbeck, has received royalties from Cambridge Cognition (CANTAB), has received editorial honoraria from Springer Verlag, Elsevier, Society for Neuroscience; has performed educational lectures for Merck, Sharpe and Dohme and does consultancy work for Cambridge Cognition, Eli Lilly, Lundbeck, Teva and Shire Pharmaceuticals. Barbara J Sahakian consults for Cambridge Cognition, PEAK and Mundipharma. William Deakin currently advises or carries out research funded by Autifony, Sunovion, Lundbeck, AstraZeneca and Servier. All payment is to the University of Manchester. Edward T Bullmore was employed half‐time by the University of Cambridge and half‐time by GSK during some of this work, and is a shareholder in GSK. Liam J Nestor was employed by GSK during some of this work. Eugenii Rabiner worked for GSK until 2011 and is a shareholder in GSK. He is a consultant to GSK, TEVA, Lightlake Therapeutics, AbbVie, and Roche.

## Author contributions

Liam J Nestor, Anna Murphy, John McGonigle, Csaba Orban, Eleanor Taylor, Remy Flechais, Louise M Paterson, Dana Smith and Karen D Ersche helped set up the study and collected the data; John McGonigle organised the study database; John McGonigle and Liam J Nestor conducted statistical analyses; Liam J Nestor wrote the first draft of the manuscript and all other authors subsequently contributed to the manuscript.

**Abbreviations**
3T: 3 tesla
ACC: anterior cingulate cortex
AIC: anterior insula cortex
ANOVA: analysis of variance
ASSIST: alcohol, smoking and substance involvement screening test
AUD: alcohol use disorder
BET: brain extraction tool
BIS‐11: barratt impulsivity scale
BOLD: blood oxygen level dependent
FEAT: fMRI expert analysis tool
FLAME: FMRIB's local analysis of mixed effects
FMRIB: functional MRI of the brain
fMRI: functional magnetic resonance imaging
FP: frontal pole
FSL: functional MRI of the brain software library
GNG: go/no‐go
IFG: inferior frontal gyrus
IQ: intelligence quotient
Log: logarithm
MCFLIRT: motion correction functional MRI of the brain linear registration tool
MNI: montreal neurological institute
MOR: mu opioid receptor
OFC: orbitofrontal cortex
PIC: participant identification centre
RIV: relative ‘impulsivity’ value
SPSS: statistical package for the social sciences
SST: stop signal task
SUD: substance use disorder
UCLH: university college London hospital
UPPS‐P: urgency, premeditation, perseverance, sensation seeking, positive urgency, impulsive behaviour scale

## Funding

The authors disclosed receipt of the following financial support for the research, authorship and/or publication of this article: This article presents independent research funded by the MRC as part of their addiction initiative (grant number G1000018). GSK kindly funded the functional and structural MRI scans that took place at the London site for this study.

## Supporting information

Fig. S1. Showing average BOLD activation changes across the whole brain for ‘stops’ during the placebo session in the AUD, poly‐SUD and control groups. Z (Gaussianised T) statistic images were thresholded using clusters determined by *Z *>* *2.3 and corrected cluster significance level of *P *<* *0.05. The scale represents the colour (from dark to light yellow) of the cluster corresponding to the increasing zt‐statistic. The structural image represents the MNI152 average normal brain with corresponding horizontal coordinates (inferior‐superior).

Fig. S2. Showing average BOLD activation changes across the whole brain for ‘stops’ during the naltrexone session in the AUD, poly‐SUD and control groups. *Z* (Gaussianised T) statistic images were thresholded using clusters determined by *Z *>* *2.3 and corrected cluster significance level of *P *<* *0.05. The scale represents the colour (from dark to light yellow) of the cluster corresponding to the increasing zt‐statistic. The structural image represents the MNI152 average normal brain with corresponding horizontal coordinates (inferior‐superior).

Table S1. Demographic variables for the control, AUD and poly‐SUD groups. *Age* * *P *<* *0.05 – AUD > poly‐SUD & control; *Edu* **P <* *0.01 ‐ poly‐SUD<control; *IQ* * *P *<* *0.05 ‐ poly‐SUD<control; *Alcohol Exposure* ***P <* *0.001 control<AUD & *P <* *0.05 ‐ poly‐SUD<AUD; *Cigarette Use* **P <* *0.01 ‐ poly‐SUD > control; *Cannabis Use* ***P <* *0.001 ‐ poly‐SUD > AUD & control. Also shown are the months of abstinence from alcohol in all three groups and additional substances of dependence in the poly‐SUD group. Data are expressed as means ± SEM. Ranges of substance asbtinence are also provided in parentheses.

## Data Availability

All relevant data are available upon request from the ICCAM consortium.
