# Supplementary material for: Naltrexone differentially modulates the neural correlates of motor impulse control in abstinent alcohol‐dependent and polysubstance‐dependent individuals
Source: Eur J Neurosci. 2018 Nov 26;50(3):2311–21. doi: 10.1111/ejn.14262 (PMC6767584; doi:10.1111/ejn.14262)
Supplement: Supplementary file 1 — Fig. S1. Showing average BOLD activation changes across the whole brain for ‘stops’ during the placebo session in the AUD, poly‐SUD and control groups. Z (Gaussianised T) statistic images were thresholded using clusters determined by Z > 2.3 and corrected cluster significance level of P < 0.05. The scale represents the colour (from dark to light yellow) of the cluster corresponding to the increasing zt‐statistic. The structural image represents the MNI152 average normal brain with corresponding horizontal coordinates (inferior‐superior). [file EJN-50-2311-s001.docx]

**Supplementary Figure 1**. Showing average BOLD activation changes across the whole brain for “stops” during the placebo session in the AUD, poly-SUD and control groups. *Z* (Gaussianized T) statistic images were thresholded using clusters determined by *Z*>2.3 and corrected cluster significance level of *p*<0.05. The scale represents the colour (from dark to light yellow) of the cluster corresponding to the increasing zt-statistic. The structural image represents the MNI152 average normal brain with corresponding horizontal coordinates (inferior-superior).
