# Supplementary material for: Naltrexone differentially modulates the neural correlates of motor impulse control in abstinent alcohol‐dependent and polysubstance‐dependent individuals
Source: Eur J Neurosci. 2018 Nov 26;50(3):2311–21. doi: 10.1111/ejn.14262 (PMC6767584; doi:10.1111/ejn.14262)
Supplement: Supplementary file 3 — Table S1. Demographic variables for the control, AUD and poly‐SUD groups. Age * P < 0.05 – AUD > poly‐SUD & control; Edu **P < 0.01 ‐ poly‐SUD<control; IQ * P < 0.05 ‐ poly‐SUD<control; Alcohol Exposure ***P < 0.001 control<AUD & *P < 0.05 ‐ poly‐SUD<AUD; Cigarette Use **P < 0.01 ‐ poly‐SUD > control; Cannabis Use ***P < 0.001 ‐ poly‐SUD > AUD & control. Also shown are the months of abstinence from alcohol in all three groups and additional substances of dependence in the poly‐SUD group. Data are expressed as means ± SEM. Ranges of substance asbtinence are also provided in parentheses. [file EJN-50-2311-s003.docx]

**Supplementary Table 1**. Demographic variables for the control, AUD and poly-SUD groups. *Age* **p*<0.05 - AUD>poly-SUD & control; *Edu* ***p*<0.01 - poly-SUD<control; *IQ* **p*<0.05 - poly-SUD<control; *Alcohol Exposure* ****p*<0.001 control<AUD & **p*<0.05 - poly-SUD<AUD; *Cigarette Use* ***p*<0.01 - poly-SUD>control; *Cannabis Use* ****p*<0.001 - poly-SUD>AUD & control. Also shown are the months of abstinence from alcohol in all three groups and additional substances of dependence in the poly-SUD group. Data are expressed as means ± SEM. Ranges of substance asbtinence are also provided in parentheses.

|  |  |  |  |
| --- | --- | --- | --- |
|  | **Control (n=35)** | **AUD (n=21)** | **Poly-SUD (n=25)** |
| **Gender (Female/Male)** | 7/28 | 4/17 | 6/19 |
| **Age** | 41.11 ± 1.54 | 46.23 ± 1.96* | 39.60 ± 1.52 |
| **Edu** | 13.45 ± 0.45 | 12.66 ± 0.65 | 11.32 ± 0.42** |
| **IQ** | 105.91 ± 1.71 | 105.28 ± 1.82 | 99.36 ± 2.39* |
| **Handedness** | 46.08 ± 9.75 | 55.74 ± 14.12 | 62.91 ± 11.22 |
| **Alcohol Exposure (yrs)** | 0.80 ± 0.44*** | 18.71 ± 1.88 | 13.42 ± 1.94* |
| **Cigarette Use (pack yrs)** | 9.99 ± 2.11 | 17.44 ± 4.45 | 22.27 ± 3.31** |
| **Cannabis Use (yrs)** | 0.34 ± 0.34 | 2.80 ± 1.05 | 8.64 ± 1.78*** |
| **Alcohol Abstinence (mths)** | 0.34 ± 0.2 (5.0) | 14.08 ± 4.23 (78.5) | 13.69 ± 2.50 (34.5) |
| **Cocaine Abstinence (mths)** | - | - | 24.10 ± 4.86 (82.5) |
| **Opiate Abstinence (mths)** | - | - | 39.47 ± 14.75 (274) |
| **Amphetamine Abstinence (mths)** | - | - | 156.85 ± 51.48 (306) |
| **Benzodiazepine Abstinence (mths)** | - | - | 64.50 ± 51.87 (161.5) |
| **GHB Abstinence (mths)** | - | - | 36.0 ± 0.00 (0) |
| **Solvent Abstinence (mths)** | - | - | 396.0 ± 0.00 (0) |
|  |  |  |  |

|  |  |  |  |
| --- | --- | --- | --- |
|  |  |  |  |
|  |  |  |  |
