# Supplementary material for: Naltrexone differentially modulates the neural correlates of motor impulse control in abstinent alcohol‐dependent and polysubstance‐dependent individuals
Source: Eur J Neurosci. 2018 Nov 26;50(3):2311–21. doi: 10.1111/ejn.14262 (PMC6767584; doi:10.1111/ejn.14262)
Supplement: Supplementary file 4 [file EJN-50-2311-s004.docx]

Naltrexone differentially modulates the neural correlates of motor impulse control in abstinent alcohol-dependent and poly-substance dependent individuals.

Nestor, Liam ; Murphy, Anna; McGonigle, John; Orban, Csaba; Reed, Laurence; Taylor, Eleanor; Flechais, Remy; Paterson, Louise; Smith, Dana; Bullmore, Ed; Ersche, Karen; Suckling, John; Elliott, Rebecca; Deakin, Bill; Rabiner, Ilan; Lingford Hughes, Anne; Sahakian, Barbara; Robbins, Trevor; Nutt, David

_____________________________________________________________________________________

| Review timeline: | Submission date: 23/03/18  Editorial Decision: Minor Revision  Revision Received: 17/08/18  Editorial Decision: Minor Revision  Revision Received: 18/09/18  Accepted: 21/09/18 |  |
| --- | --- | --- |
|  |  |  |

Editor: Rita Goldstein

Reviewer 1: Chiang-Shan

Reviewer 2: Corinde Wiers

_____________________________________________________________________________________

1st Editorial Decision	07/08/18

_____________________________________________________________________________________

Naltrexone differentially modulates the neural correlates of motor impulse control in abstinent alcoholic and poly substance abusers.

Dear Dr. Nestor,

Your paper has now been reviewed by two external experts and our guest Section Editor, Prof. Goldstein. We sincerely apologize for the inordinately long time the review process has taken – this is highly atypical here at EJN. Both reviewers see significant merit in the work, although they have also asked for changes and additions to the text and analyses that should strengthen this already very solid paper. As such, we are returning the paper to you for a round of revisions. As you will read, there is a valuable suggestion for additional analysis of the behavioral data to potentially adjudicate between an impulse control or working memory account. Also, there is a call for a more elaborated discussion of aspects of the extant literature and a more detailed exposition of potential molecular mechanisms.  Please also make clear the rationale behind the choice of experimental cohorts, and more phenotyping details would also be welcome. We find that most of the criticisms should be relatively easy to answer in a revision and look forward to receiving your manuscript again in short order.

Please also attend to the following issues identified by our editorial office:

1. Author contributions need to be explicitly included in a statement in the manuscript itself
2. An Abbreviation list is needed
3. You will need to explicitly state that ethical approval for the study was received and name the board that oversaw the project. Are your procedures consistent with the declaration of Helsinki?
4. In your statistics, P values are reported as inequalities. Please report exact values.
5. We will not proceed until you replace your barcharts with scatterplots or some other more informative hybrid depictions - see http://onlinelibrary.wiley.com/doi/10.1111/ejn.13400/epdf
6. Supplementary table needs to be clearly labelled as such (not just as 'table 1')

When revising the manuscript, please embolden or underline major changes to the text so they are easily identifiable and DO NOT leave 'track change' formatting marks in your paper.  Please ensure that you provide a text and a figure file for the Graphical Abstract (as detailed in the instructions below).  When carrying out your revisions please refer to the checklist below and visit the EJN author guidelines at www.ejneuroscience.org

When finalized, please upload your complete revised manuscript onto the website, as a Word file (.doc, or .docx).  Please also ensure that a complete set of tables and figures is included as separate files, even if these have not changed from the originals.  At this stage it is necessary to provide high resolution figures.  Please see important instructions below.

Please go into https://mc.manuscriptcentral.com/ejn - Author Centre - manuscripts with decisions where you will find a 'create a revision' link under 'actions'. We ask that you please indicate the way in which you have responded to the points raised by the Editors and Reviewers in a letter. Please upload this response letter as a separate Word (.doc or PDF) file using the file designation "Authors' Response to Reviewers" when uploading your manuscript files.  Please DO NOT submit your revised manuscript as a new one.  Also, please note that only the  Author who submitted the original version of the manuscript should submit a revised version.

If you are able to respond fully to the points raised, we would be pleased to receive a revision of your paper within 30 days.

Thank you for submitting your work to EJN.

Kind regards,

John Foxe & Paul Bolam
co-Editors in Chief, EJN


Reviews:

Reviewer: 1

Comments to the Author
In a placebo-controlled double blind study, authors examined the effects of acute naltrexone (50 mg) on the neural processes of impulse control in abstinent alcohol and polysubstance users. The results showed different modulation effects in alcohol and polysubstance users. Overall, following authors’ earlier work, the study was well motivated. Experimental design and data analyses were sound, and the findings support the conclusions. I have a few comments authors may wish to consider in a revision.
Introduction: Would it be possible to build on animal work and elaborate on the potential molecular processes whereby naltrexone enhances impulse control?
Methods/Results
H. Garavan’s “XY GNG” task (2003) was employed in the current study. This paradigm requires working memory (as with “1-back”) for participants to perform accurately. An analysis to help figure out whether nogo error related more to impulsivity or to working memory is to contrast the go RT and nogo error RT. A significantly shorter nogo error RT would strongly suggest deficits in impulse control.
Imaging results were examined with a voxel Z=2.3 plus a corrected cluster threshold. A Z=2.3 is quite liberal. Have authors tried a more stringent threshold?
Discussion:
I feel the discussion of the alcohol impulse control literature is somewhat limited. This literature shows mixed result in terms of deficits in impulse control and brain responses to impulse control (e.g., Li et al., 2009 ACER; Hu et al., 2015 NIC; Hu et al., 2016 ACER; Ide et al. 2018, BPCNN). Readers would benefit from a more thorough discussion of these studies.
Minor:
Last sentence of the Abstract perhaps needs to be revised, as prediction by impulsivity trait is true only of the polysubstance group.


Reviewer: 2

Comments to the Author
This is a well-conducted and well-analyzed study on the effects of naltrexone on neural correlates of GONOGO task performance in AUD, poly-substance use disorder and healthy controls. I have few suggestions for improvement:

(1) The title doesn’t read well: what is an (abstinent) alcoholic […] abuser? I would recommend the clinical diagnostic terminology, e.g., “abstinent individuals with alcohol – and poly substance use disorder.”, or, since you diagnosed with DSM-IV: “abstinent alcohol-dependent and poly-substance dependent individuals”

(2) It is unclear to me why AUD and poly-SUD are included as separate groups. What are the specific hypotheses comparing these 2 groups? And if there are none (for example, the introduction only specifies hypotheses of both SUD groups compared to controls), then why not pool them together rather than running an ANOVA. Alternatively, provide expectations on between-group contrasts.

(3) The group names are slightly confusing: To me, Alcoholminus intuitively means the same as controls, or perhaps AUD subjects who are abstinent. I understand the reason for naming the two SUD groups Alcoholminus and Alcoholplus, but I would nevertheless recommend to use AUD and poly-SUD, for example, to not confuse readers.

(4) Methods: Without having to refer to Nestor et al (2017): what was the range of abstinence of alcohol and other drugs?  Did the two SUD groups differ in abstinence or severity of dependence? It is presented in Table 1 (not Supplemental Table 1, as mentioned on Page 10?), but it would be good to include the statement in the main manuscript that dependent groups did not differ in alcohol abstinence, or cigarette use, but Alcoholminus had a longer history of alcohol exposure. Were group comparisons corrected for this? Also refer to this Table 1in “Participants” section.

(5) Methods: What is “ASSIST”? How were clinical diagnoses assessed? SCID or MINI? Was this done by a psychiatrist?

(6) Results: provide exact statements on demographic and clinical differences between groups.

(7) Results: The 3x2 analyses for behavior and functional MRI are sound. However, it may be helpful to first analyze cases vs controls; particularly if there are no a priori expected group differences between case groups.

(8) The discussion is very clear and concise. Could the findings be associated with decreased GMV in abstinent AUD participants in frontal cortex (e.g., Fein ea 2002 ACER, Grodin ea 2013 Neuroimage Clin, Trick ea 2014 Addiction Biology, Gropper ea J Psychiatric Res 2016)? There is a wide literature on that, including associations with inhibitory control. Moreover, acute alcohol exposure showed lower fronto-temporal cortex activation during an inhibitory controls task (Gan ea 2014 Biol Psychiatry).

(9) Figure 1D: was there a trend effect of group on mean error reaction times? The alcohol minus group seems lower for both Naltrexone and Placebo, compared to controls and + group

------------------------

____________________________________________________________________________________

Authors’ Response	17/08/18

_____________________________________________________________________________________

07-Aug-2018

Manuscript Number:EJN-2018-03-25441(ADX) Naltrexone differentially modulates the neural correlates of motor impulse control in abstinent alcoholic and poly substance abusers.

Dear Dr. Nestor,

Your paper has now been reviewed by two external experts and our guest Section Editor, Prof. Goldstein. We sincerely apologize for the inordinately long time the review process has taken – this is highly atypical here at EJN. Both reviewers see significant merit in the work, although they have also asked for changes and additions to the text and analyses that should strengthen this already very solid paper. As such, we are returning the paper to you for a round of revisions. As you will read, there is a valuable suggestion for additional analysis of the behavioral data to potentially adjudicate between an impulse control or working memory account. Also, there is a call for a more elaborated discussion of aspects of the extant literature and a more detailed exposition of potential molecular mechanisms. Please also make clear the rationale behind the choice of experimental cohorts, and more phenotyping details would also be welcome. We find that most of the criticisms should be relatively easy to answer in a revision and look forward to receiving your manuscript again in short order.

Please also attend to the following issues identified by our editorial office:

1. Author contributions need to be explicitly included in a statement in the manuscript itself

**Author response**: This has now been inserted at the end of the manuscript (under Contribution Statement) as follows:

**Liam J Nestor, Anna Murphy, John McGonigle, Csaba Orban, Eleanor Taylor, Remy Flechais, Louise M Paterson, Dana Smith and Karen D Ersche helped set up the study and collected the data; John McGonigle organized the study database; John McGonigle and Liam J Nestor conducted statistical analyses; Liam J Nestor wrote the first draft of the manuscript and all other authors subsequently contributed to the manuscript.**

2. An Abbreviation list is needed

**Author response**: This has now been inserted at the end of the manuscript.

3. You will need to explicitly state that ethical approval for the study was received and name the board that oversaw the project. Are your procedures consistent with the declaration of Helsinki?

**Author response**: Ethical approval was received to conduct the study. We have now made a statement to this effect in the manuscript (under Participants) as follows:

**The study was conducted in accordance with the Declaration of Helsinki. Ethical approval was obtained from West London and Gene Therapy Advisory Committee National Research Ethics Service committee (11/H0707/9) and relevant research governance and Participant Identification Centre (PIC) approvals obtained.**

4. In your statistics, P values are reported as inequalities. Please report exact values.

**Author response**: Exact p values, as opposed to inequalities, have now been reported.

5. We will not proceed until you replace your barcharts with scatterplots or some other more informative hybrid depictions - see <http://onlinelibrary.wiley.com/doi/10.1111/ejn.13400/epdf>

**Author response**: We have now replaced all bar charts with scatterplots.

6. Supplementary table needs to be clearly labelled as such (not just as 'table 1')

**Author response**: Table 1 has now been labelled as supplementary table 1.

When revising the manuscript, please embolden or underline major changes to the text so they are easily identifiable and DO NOT leave 'track change' formatting marks in your paper. Please ensure that you provide a text and a figure file for the Graphical Abstract (as detailed in the instructions below). When carrying out your revisions please refer to the checklist below and visit the EJN author guidelines at [www.ejneuroscience.org](http://www.ejneuroscience.org)

When finalized, please upload your complete revised manuscript onto the website, as a Word file (.doc, or .docx). Please also ensure that a complete set of tables and figures is included as separate files, even if these have not changed from the originals. At this stage it is necessary to provide high resolution figures. Please see important instructions below.

Please go into <https://mc.manuscriptcentral.com/ejn> - Author Centre - manuscripts with decisions where you will find a 'create a revision' link under 'actions'. We ask that you please indicate the way in which you have responded to the points raised by the Editors and Reviewers in a letter. Please upload this response letter as a separate Word (.doc or PDF) file using the file designation "Authors' Response to Reviewers" when uploading your manuscript files. Please DO NOT submit your revised manuscript as a new one. Also, please note that only the Author who submitted the original version of the manuscript should submit a revised version.

If you are able to respond fully to the points raised, we would be pleased to receive a revision of your paper within 30 days.

Thank you for submitting your work to EJN.

Kind regards,

John Foxe & Paul Bolam

co-Editors in Chief, EJN

Reviews:

Reviewer: 1

Comments to the Author

In a placebo-controlled double blind study, authors examined the effects of acute naltrexone (50 mg) on the neural processes of impulse control in abstinent alcohol and polysubstance users. The results showed different modulation effects in alcohol and polysubstance users. Overall, following authors’ earlier work, the study was well motivated. Experimental design and data analyses were sound, and the findings support the conclusions. I have a few comments authors may wish to consider in a revision.

Introduction: Would it be possible to build on animal work and elaborate on the potential molecular processes whereby naltrexone enhances impulse control?

**Author response**: We have now added this suggestion into the introduction (second paragraph) as follows:

**Pre-clinical animal models show that the mu opioid receptor (MOR) plays a significant role in addiction (Giuliano et al., 2013), particularly with respect to alcohol-seeking and consumption (Boyle et al., 1998; Dayas et al., 2007; Giuliano et al., 2015). Importantly, appetitive motivation for addictive substances may also occur due to disturbances in the prefrontal opioid system that could conceivably result in the recruitment of diverse and functionally opposed pathways (Baldo, 2016) that lead to a breakdown in mechanisms of inhibitory control, which promote substance relapse. Indeed, animal models have shown that the MOR promotes impulsive behaviours that are actually dissociated from alcohol or drug consumption (Mahoney et al., 2013; Olmstead et al., 2009; Pattij et al., 2009; Sanchez-Roige et al., 2015), suggesting that opioid disturbances within prefrontal brain networks could be targets for diminishing impulsivity in addiction disorders.**

Methods/Results

H. Garavan’s “XY GNG” task (2003) was employed in the current study. This paradigm requires working memory (as with “1-back”) for participants to perform accurately. An analysis to help figure out whether nogo error related more to impulsivity or to working memory is to contrast the go RT and nogo error RT. A significantly shorter nogo error RT would strongly suggest deficits in impulse control.

**Author response**: We have now computed and analysed an “index” to evaluate this using reaction time (rt) on the “go” and “error” trials. This was done by dividing the “go” rt by the “error” rt (“go” rt/“error” rt) to produce a relative “impulsivity” value for each participant in each group. Here a value >1 reflects a higher relative value of impulsive responding when a participant commits an “error” on a no-go trial. We have then conducted a three x repeated measures ANOVA on this index. We have now inserted text to describe this in the methods section (under Other Statistics*)* as follows:

**We also conducted a three (Group: AUD versus poly-SUD versus control) by two (Drug: placebo versus naltrexone) repeated measures ANOVA on an index of the relative “impulsivity” value (RIV). This value is based on the ratio of mean reaction times on “go” trials compared to that on “error” trials—i.e. (“go” rt/“error” rt. Here a value >1 reflects a higher relative value of impulsive responding when a participant commits an “error” on a no-go trial.**

We have also inserted additional text into the results section (under GNG Performance*)* as follows*:*

**Finally, for the RIV index, there was no effect of drug (*F*=1.06; df=1, 78; *p*=0.31), group (*F*=1.93; df=2, 78; *p*=0.15), or a drug x group interaction (*F*=0.44; df=2, 78; *p=*0.64 - Fig 1E).**

Imaging results were examined with a voxel Z=2.3 plus a corrected cluster threshold. A Z=2.3 is quite liberal. Have authors tried a more stringent threshold?

**Author response**: We did try a more conservative threshold of Z>3.1 in FSL, but many of the effects did not survive.

Discussion:

I feel the discussion of the alcohol impulse control literature is somewhat limited. This literature shows mixed result in terms of deficits in impulse control and brain responses to impulse control (e.g., Li et al., 2009 ACER; Hu et al., 2015 NIC; Hu et al., 2016 ACER; Ide et al. 2018, BPCNN). Readers would benefit from a more thorough discussion of these studies.

**Author response**: We have now added this suggestion into the discussion as follows:

**Addiction disorders, such as AUD, are generally associated with impairments in impulse control (Smith et al., 2014). There is a mixed literature regarding the neural correlates of impulse control in AUD, however. The reported activation pattern observed in the FP of the AUD group in the present study, for example, may concur with previous results in abstinent alcoholics during impulse control (Li et al., 2009), who similarly found increased activation in the bilateral prefrontal cortex using a stop signal task (SST). Another study has shown greater cortical activation patterns in AUD (Hu et al., 2015), but not in the prefrontal cortex, and in the presence of poorer impulse control. Other studies, however, have not reported group differences on the neural substrates of impulse control in AUD, instead showing associations between diminished prefrontal responses and harmful alcohol use (Hu et al., 2016) or no differences at all (Taylor et al., 2016), despite high self-report measures of impulsivity. These differences in impulse control and its neural correlates in AUD across studies may reflect contrasting durations of abstinence at the time of testing that represent differences in the remission of impulsivity in AUD (Naim-Feil et al., 2014; Sinha, 2011).**

Minor:

Last sentence of the Abstract perhaps needs to be revised, as prediction by impulsivity trait is true only of the polysubstance group.

**Author response**: We have now changed this to make it clear that trait impulsivity predicted the response to naltrexone in only the poly-SUD group as follows:

**…which are predicted by trait impulsivity in the poly substance dependent group.**

Reviewer: 2

Comments to the Author

This is a well-conducted and well-analyzed study on the effects of naltrexone on neural correlates of GONOGO task performance in AUD, poly-substance use disorder and healthy controls. I have few suggestions for improvement:

(1) The title doesn’t read well: what is an (abstinent) alcoholic […] abuser? I would recommend the clinical diagnostic terminology, e.g., “abstinent individuals with alcohol – and poly substance use disorder.”, or, since you diagnosed with DSM-IV: “abstinent alcohol-dependent and poly-substance dependent individuals”

**Author response**: We have now changed the title to:

**Naltrexone differentially modulates the neural correlates of motor impulse control in abstinent alcohol-dependent and poly-substance dependent individuals.**

(2) It is unclear to me why AUD and poly-SUD are included as separate groups. What are the specific hypotheses comparing these 2 groups? And if there are none (for example, the introduction only specifies hypotheses of both SUD groups compared to controls), then why not pool them together rather than running an ANOVA. Alternatively, provide expectations on between-group contrasts.

**Author response**: We chose to “phenotype” the groups due to clear differences in illicit substance addiction - the alcohol_plus_ group were diagnosed by a psychiatrist with additional dependencies to alcohol. We did not make a clear prediction about how the two addiction groups would differ from the control group. By conflating the two addiction groups we will eliminate, what we believe, are the most significant findings of the study involving the interactions, which are driven by differences on naltrexone in the two addiction groups. Therefore, we have now re-phrased the last paragraph of the introduction and our hypotheses as follows:

**Comorbid alcohol and drug dependence may be particularly detrimental to health. Individuals who have been dependent on multiples substances report the consumption of more alcohol, and have a greater incidence and severity of psychiatric illness, than individuals who are exclusively alcohol-dependent (Moss et al., 2015). This divergence in abuse and dependence, could conceivably, induce disparate neural responses that are a product of dependence “phenotype”, and that differentially respond to medications that modulate endogenous opioid functioning. Therefore, the current study investigated the effects of acute MOR blockade with naltrexone on the neural correlates of motor impulse control using a go/no-go task (GNG) task in alcoholic *and* poly substance-dependent individuals who were in extended abstinence. The GNG task is sensitive to the neural correlates of impulse control during heightened performance monitoring, exploiting the same prefrontal networks that are targets of the endogenous opioid system and MOR blockade with naltrexone. Therefore, we hypothesized that compared with controls, alcoholic and poly substance-dependent groups would 1) show poorer impulse control, 2) demonstrate disparate disturbances within prefrontal networks due to dependence “phenotype”, and 3) demonstrate a dissimilar remediating effect on neural disturbances following acute MOR blockade with naltrexone.**

(3) The group names are slightly confusing: To me, Alcoholminus intuitively means the same as controls, or perhaps AUD subjects who are abstinent. I understand the reason for naming the two SUD groups Alcoholminus and Alcoholplus, but I would nevertheless recommend to use AUD and poly-SUD, for example, to not confuse readers.

**Author response**: We have changed the alcohol and poly groups to AUD and poly-SUD, respectively.

(4) Methods: Without having to refer to Nestor et al (2017): what was the range of abstinence of alcohol and other drugs? Did the two SUD groups differ in abstinence or severity of dependence? It is presented in Table 1 (not Supplemental Table 1, as mentioned on Page 10?), but it would be good to include the statement in the main manuscript that dependent groups did not differ in alcohol abstinence, or cigarette use, but Alcoholminus had a longer history of alcohol exposure. Were group comparisons corrected for this? Also refer to this Table 1in “Participants” section.

**Author response**: We have now included a statement to this effect in the results section (under Demographics) as follows:

**The AUD and poly-SUD groups, however, did not significantly differ on cigarette use (pack years) or alcohol abstinence, but the AUD group did have significantly more alcohol exposure than the poly-SUD group.**

(5) Methods: What is “ASSIST”? How were clinical diagnoses assessed? SCID or MINI? Was this done by a psychiatrist?

**Author response**: The ASSIST is the Alcohol, Smoking and Substance Involvement Screening Test (ASSIST). The Mini*-*International Neuropsychiatric Interview *(*MINI*)* was conducted on all participants by a trained psychiatrist.

(6) Results: provide exact statements on demographic and clinical differences between groups.

**Author response**: We have now provided statements to this effect in the results section (under Demographics) as follows:

**The groups significantly differed on most of the measures reported (see Table 1 in supplementary materials for a more detailed description of demographic group differences). The AUD group was significantly older than the poly-SUD group; the poly-SUD group reported significantly less years of education, and had a lower estimated IQ, than the control group; the poly-SUD group had significantly more cigarette use than the control group, and reported significantly more cannabis use than both controls and the poly-SUD group. The AUD and poly-SUD groups, however, did not significantly differ on cigarette use (pack years) or alcohol abstinence, but the AUD group did have significantly more alcohol exposure than the poly-SUD group. Importantly, the three groups did not differ significantly on drug treatment order (χ^2^ = 0.48, df=2, p= 0.78) during the study.**

(7) Results: The 3x2 analyses for behavior and functional MRI are sound. However, it may be helpful to first analyze cases vs controls; particularly if there are no a priori expected group differences between case groups.

**Author response**: Please see response to number (2) above.

(8) The discussion is very clear and concise. Could the findings be associated with decreased GMV in abstinent AUD participants in frontal cortex (e.g., Fein ea 2002 ACER, Grodin ea 2013 Neuroimage Clin, Trick ea 2014 Addiction Biology, Gropper ea J Psychiatric Res 2016)? There is a wide literature on that, including associations with inhibitory control. Moreover, acute alcohol exposure showed lower fronto-temporal cortex activation during an inhibitory controls task (Gan ea 2014 Biol Psychiatry).

**Author response**: We have now discussed this as a possibility in the discussion as follows:

**Another possibility is that the greater activation patterns observed in the AUD group represent deficits in cortical grey matter volume (GMV) in the medial and lateral prefrontal regions. There is evidence that patients with AUD have density (Thayer et al., 2016) and volume deficits (Gropper et al., 2016; Medina et al., 2008; Trick et al., 2014; Xiao et al., 2015) in the prefrontal cortex, which may also be correlated with impulse control (Grodin et al., 2017; Gropper et al., 2016; Wiers et al., 2015), and predict future relapse (Cardenas et al., 2011). This raises the possibility that the effects observed in the AUD group represent some compensatory mechanism by which prefrontal networks expend excessive energy to support cognitive control processes in abstinence due to structural brain deficits caused by alcohol abuse. The correlation between alcohol abstinence and FP/IFG activation in the AUD group, however, may discount this possibility.**

(9) Figure 1D: was there a trend effect of group on mean error reaction times? The alcohol minus group seems lower for both Naltrexone and Placebo, compared to controls and + group

**Author response**: There was indeed a trend. We have since attempted to evaluate this effect as per the request of reviewer 1. Therefore, we also conducted a three (Group: AUD versus poly-SUD versus control) by two (Drug: placebo versus naltrexone) repeated measures ANOVA on an index of the relative “impulsivity” value (RIV). This value is based on the ratio of mean reaction times on “go” trials compared to that on “error” trials—i.e. (“go” rt/“error” rt. Here a value >1 reflects a higher relative value of impulsive responding when a participant commits an “error” on a no-go trial. There were no significant findings on this index, however.

2^nd^ Editorial Decision	18/09/18

_____________________________________________________________________________________

Naltrexone differentially modulates the neural correlates of motor impulse control in abstinent alcohol-dependent and poly-substance dependent individuals.

Dear Dr. Nestor,

Your revised manuscript was re-evaluated by the two original external reviewers as well as by the Section Editor, Dr. Rita Goldstein and ourselves.  We are pleased to inform you that we expect that it will be acceptable for publication in EJN following a further quick round of minor revisions. While reviewer #1 is completely satisfied with your responses to his initial queries, reviewer #2 has just a few remaining points that need to be dealt with.  Please attend to these points as forthrightly as you can (see below).

Also, can you please provide more detail about the consenting process -- was it verbal or written/

When revising the manuscript, please embolden or underline major changes to the text so they are easily identifiable and DO NOT leave 'track change' formatting marks in your paper.  At this stage, please provide text and a figure file for the Graphical Abstract.  When carrying out your revisions please refer to the checklist below and visit the EJN author guidelines at www.ejneuroscience.org

When finalised, please upload your complete revised manuscript onto the website as a Word (.doc or .docx), or .rtf file.  Please also ensure that a complete set of tables and figures is included as separate files, even if these have not changed from the originals.  At this stage it is necessary to provide high resolution figures.  Please see important instructions below.

Please go into https://mc.manuscriptcentral.com/ejn - Author Centre - manuscripts with decisions where you will find a 'create a revision' link under 'actions'. We ask that you please indicate the way in which you have responded to the points raised by the Editors and Reviewers in a letter. Please upload this response letter as a separate Word (.doc) file using the file designation "Authors' Response to Reviewers" when uploading your manuscript files. Please DO NOT submit your revised manuscript as a new one.  Also, please note that only the  Author who submitted the original version of the manuscript should submit a revised version.

If you are able to respond fully to the points raised, we shall be pleased to receive a revision of your paper within 30 days.

Thank you for submitting your work to EJN.

Kind regards,

John Foxe & Paul Bolam
co-Editors in Chief, EJN

Reviews:

Reviewer: 1

Comments to the Author
Authors have addressed the suggestions raised in earlier review. I have no further comments. The study represents a significant addition to the literature of addiction neuroscience.

Reviewer: 2

Comments to the Author
The authors have responded very adequately to my comments.

A few minor concerns that remain:

In response to (2): The Results and Discussion mention AUD vs poly substance contrasts. However, hypotheses for these comparisons are still lacking in the Introduction. The study is unique in that it can compare these relatively well-matched groups of substance abusers.

In response to (5): “ASSIST” still needs to be spelled throughout in the text (depending on journal’s policy) and needs a reference. Also, MINI needs to be mentioned and referenced in the text; it is missing from the Methods.

In response to (6): Could neuroimaging findings be due to demographic group differences (e.g., age, education and IQ)? If, despite demographic group differences, no covariates were added to analyses please elaborate in Discussion.

_____________________________________________________________________________________

Authors’ Response	10/09/18

_____________________________________________________________________________________

correlates of motor impulse control in abstinent alcohol-dependent and poly-substance dependent individuals.

Dear Dr. Nestor,

Your revised manuscript was re-evaluated by the two original external reviewers as well as by the Section Editor, Dr. Rita Goldstein and ourselves. We are pleased to inform you that we expect that it will be acceptable for publication in EJN following a further quick round of minor revisions. While reviewer #1 is completely satisfied with your responses to his initial queries, reviewer #2 has just a few remaining points that need to be dealt with. Please attend to these points as forthrightly as you can (see below).

Also, can you please provide more detail about the consenting process -- was it verbal or written/

**Author response**: The consenting process was written. We have now provided a short statement to this effect at the beginning of the last paragraph (under Participants) as follows:

**All participants provided written informed consent.**

When revising the manuscript, please embolden or underline major changes to the text so they are easily identifiable and DO NOT leave 'track change' formatting marks in your paper. At this stage, please provide text and a figure file for the Graphical Abstract. When carrying out your revisions please refer to the checklist below and visit the EJN author guidelines at [www.ejneuroscience.org](http://www.ejneuroscience.org)

When finalised, please upload your complete revised manuscript onto the website as a Word (.doc or .docx), or .rtf file. Please also ensure that a complete set of tables and figures is included as separate files, even if these have not changed from the originals. At this stage it is necessary to provide high resolution figures. Please see important instructions below.

Please go into <https://mc.manuscriptcentral.com/ejn> - Author Centre - manuscripts with decisions where you will find a 'create a revision' link under 'actions'. We ask that you please indicate the way in which you have responded to the points raised by the Editors and Reviewers in a letter. Please upload this response letter as a separate Word (.doc) file using the file designation "Authors' Response to Reviewers" when uploading your manuscript files. Please DO NOT submit your revised manuscript as a new one. Also, please note that only the Author who submitted the original version of the manuscript should submit a revised version.

If you are able to respond fully to the points raised, we shall be pleased to receive a revision of your paper within 30 days.

Thank you for submitting your work to EJN.

Kind regards,

John Foxe & Paul Bolam

co-Editors in Chief, EJN

Reviews:

Reviewer: 1

Comments to the Author

Authors have addressed the suggestions raised in earlier review. I have no further comments. The study represents a significant addition to the literature of addiction neuroscience.

Reviewer: 2

Comments to the Author

The authors have responded very adequately to my comments.

A few minor concerns that remain:

In response to (2): The Results and Discussion mention AUD vs poly substance contrasts. However, hypotheses for these comparisons are still lacking in the Introduction. The study is unique in that it can compare these relatively well-matched groups of substance abusers.

**Author response**: We have now rephrased the hypotheses as follows:

**Therefore, we hypothesized that 1) compared to the control group, the alcoholic and poly substance-dependent groups would show poorer motor impulse control, 2) compared to the control group, the alcoholic and poly substance-dependent groups would demonstrate disparate functional differences within prefrontal networks due to dependence “phenotype”, and 3) that the alcoholic and poly substance-dependent groups would demonstrate disparate remediating effects on these functional differences following acute MOR blockade with naltrexone.**

In response to (5): “ASSIST” still needs to be spelled throughout in the text (depending on journal’s policy) and needs a reference. Also, MINI needs to be mentioned and referenced in the text; it is missing from the Methods.

**Author response**: We have now fully mentioned and referenced the ASSIST and MINI as follows:

**The healthy control group was made up of 35 participants with no previous history of substance abuse, as assessed using the Alcohol, Smoking and Substance Involvement Screening Test (ASSIST) (WHO ASSIST Working Group, 2002) and timeline follow-back.**

**The Mini*-*International Neuropsychiatric Interview *(*MINI) (Sheehan et al., 1998) was administered to all participants by a trained psychiatrist to screen for the presence of Axis I psychiatric disorders that were part of the study exclusion criteria.**

In response to (6): Could neuroimaging findings be due to demographic group differences (e.g., age, education and IQ)? If, despite demographic group differences, no covariates were added to analyses please elaborate in Discussion.

**Author response**: We have now further mentioned these differences in a limitations paragraph (penultimate paragraph of the Discussion) and reported that we did not use them as covariates in any of our fMRI analyses. We have rephrased this statement as follows:

**There were a number of limitations of the current study, which included a lack of complete matching of groups with respect to age, cannabis and cigarette use, anxiety and mood measures. We did not use these metrics as covariates for any of our fMRI analyses, and therefore, cannot unequivocally dismiss their potential influence on the group and interaction effects reported herein.**

------------------------
